# Tissue tropisms, infection kinetics, histologic lesions, and antibody response of the MR766 strain of Zika virus in a murine model

**DOI:** 10.1186/s12985-017-0749-x

**Published:** 2017-04-18

**Authors:** Anna B. Kawiecki, E. Handly Mayton, M. Fausta Dutuze, Brad A. Goupil, Ingeborg M. Langohr, Fabio Del Piero, Rebecca C. Christofferson

**Affiliations:** Department of Pathobiological Sciences, School of Veterinary Medicine, Louisiana State University, Baton Rouge, LA USA

**Keywords:** Zika virus, Mouse model, Neutralizing antibody, Pathogenesis, Histology, Immunohistochemistry

## Abstract

**Background:**

The appearance of severe Zika virus (ZIKV) disease in the most recent outbreak has prompted researchers to respond through the development of tools to quickly characterize transmission and pathology. We describe here another such tool, a mouse model of ZIKV infection and pathogenesis using the MR766 strain of virus that adds to the growing body of knowledge regarding ZIKV kinetics in small animal models.

**Methods:**

We infected mice with the MR766 strain of ZIKV to determine infection kinetics via serum viremia. We further evaluated infection-induced lesions via histopathology and visualized viral antigen via immunohistochemical labeling. We also investigated the antibody response of recovered animals to both the MR766 and a strain from the current outbreak (PRVABC59).

**Results:**

We demonstrate that the IRF3/7 DKO mouse is a susceptible, mostly non-lethal model well suited for the study of infection kinetics, pathological progression, and antibody response. Infected mice presented lesions in tissues that have been associated with ZIKV infection in the human population, such as the eyes, male gonads, and central nervous system. In addition, we demonstrate that infection with the MR766 strain produces cross-neutralizing antibodies to the PRVABC59 strain of the Asian lineage.

**Conclusions:**

This model provides an additional tool for future studies into the transmission routes of ZIKV, as well as for the development of antivirals and other therapeutics, and should be included in the growing list of available tools for investigations of ZIKV infection and pathogenesis.

**Electronic supplementary material:**

The online version of this article (doi:10.1186/s12985-017-0749-x) contains supplementary material, which is available to authorized users.

## Background

Recent and rapid development of Zika virus (ZIKV) mouse models have already led to important discoveries, especially concerning congenital transmission and outcomes of ZIKV infection [1–5]. These models include interferon (IFN) type I and/or type II knockout strains, as these mice are more susceptible to many related flaviviruses compared to immunocompetent strains [1, 2, 5–7]. These models have collectively demonstrated that ZIKV causes high viral titers in mice, and have recapitulated some of the neurological, congenital, and ocular symptoms associated with the virus in the current outbreak.

When ZIKV recently emerged in the Western Hemisphere it was linked to congenital malformations including microcephaly, abnormal ocular development, and other neurological sequelae in both infants and adults [8–10]. In addition, retrospective analysis of a previous outbreak in French Polynesia involving the Asian lineage has suggested that these disease manifestations were also associated with that outbreak [11]. However, these congenital and neurological presentations have not been historically reported with ZIKV outbreaks in Africa where seroconversion has been demonstrated since the 1950s and which were likely associated with the African lineage [12–15]. Moreover, strains of the African lineage have been significantly less studied than those of the Asian lineage linked to the most recent outbreaks, despite evidence of continued circulation of ZIKV in Africa [15–17]. To that end, we infected a strain of C57Bl/6 mouse that lacks IFN regulatory factors (IRF) 3 and 7 with the Ugandan strain (MR766) of ZIKV to describe the pathogenesis of this prototypical strain and whether infection with MR766 elicits antibodies that cross-neutralize a strain from the current outbreak (PRVABC59).

## Methods

### Ethics statement, virus, and mouse strains

All experiments involving mice were approved by the Louisiana State University (LSU) Institutional Animal Care and Use Committee (IACUC protocol 15-078) in adherence with policies of the American Veterinary Medical Association and in compliance with the guidelines laid out by the National Institutes of Health’s Guide for Care and Use of Laboratory Animals, 2011.

The IRF3/7 double knockout (DKO) mice were originally provided by Dr. Michael Diamond and are deficient in IRF 3 and 7. These mice have a blunted but not abrogated type I interferon response [18]. The prototypical ZIKV strain MR766 was used. Virus was originally obtained from Dr. Robert Tesh at the World Reference Center for Emerging Viruses and Arboviruses at the University of Texas Medical Branch as lyophilized stock and was passaged it once in C6/36 cells and once in Vero cells prior to use in this study. MR766 was originally isolated from the Zika forest in Uganda in the late 1940s and belongs to the African genotype. For post-infection challenge, the PRVABC59 (Asian lineage) was originally received from the CDC (Ft. Collins, CO) and was passaged once on Vero cells prior to our additional passage on Vero cells. Both strains of ZIKV were determined to have a titer of ~10^7^ plaque forming units (PFU)/mL via plaque assay prior to beginning the experiments. Mice were infected by injection subcutaneously in the back with 10^6^ PFU in 100 μl.

### Experimental design

#### Characterization of infection kinetics

To characterize ZIKV infection kinetics, one group of male mice (*n* = 5) and one group of female mice (*n* = 6) were challenged with ZIKV MR766. These groups of 6- to 10-week-old mice were inoculated with 10^6^ PFU in 100 μl of ZIKV MR766 subcutaneously. Mice were bled on 1, 2, 4, and 6 days post infection (dpi). Mice were weighed on days 0, 4, 6, and 8–12 and percent reduction in weight from day 0 was calculated. If an individual lost more than 20% of the initial body weight, it was euthanized as per the approved IACUC protocol. Two individuals presented with ocular disease in the form of discharge and crusts; in these cases the discharge was collected with a sterile swab and subsequently tested for the presence of viral nucleic acid and replicating virus.

#### Histology and immunohistochemistry

Two mice from the infection kinetics study that died or were euthanized (one male on 7dpi and one female on 8 dpi) were processed for histology and immunohistochemistry. In addition, to further investigate lesions, especially of the female reproductive system, a separate group of female mice (*n* = 5) was infected and processed for histology and immunohistochemistry following confirmation of infection by qRT-PCR (see below). Following a complete multisystemic gross examination immediately after euthanasia at 7, 10 and 12 dpi (1, 2 and 2 mice, respectively), tissues were collected and fixed in 10% neutral buffered formalin. Tissues were progressively dehydrated in alcohol solutions and xylene, were embedded in paraffin, and 5 μm thick tissue sections were obtained for slide preparation, were stained with hematoxylin and eosin and coverslipped. For IHC, the slides were prepared as described previously [19], with the specific reagents as follows: mouse-on-mouse (MOM) kit for mouse primary antibody detection (Vector Labs Cat # PK-2200), control mouse IgG (Biocare, NC494H), and primary antibody 4G2 (Anti-Flavivirus group antigen antibody, EMD Millipore) at a working dilution of 1:100. The slides were examined via light microscopy and pathologic changes recorded by ACVP board certified pathologists at the LSU School of Veterinary Medicine.

#### Heterotypic challenge for investigations of antibody response

A separate group of female mice (*n* = 7) 8–10 weeks old were infected with 10^6^ PFU/mouse in a volume of 100 μl of ZIKV MR766. Detection of viral RNA was performed as described below. We opted to bleed mice every other day in a staggered design so that no mouse was bled more than allowable, but the entirety of the viremia curve was captured as described previously [6]. Four mice were bled days 1, 3, 5, and 7 day post infection (dpi) while three mice were bled 2, 4, 6, and 8 dpi. Mice were weighed just before bleeding and weight loss monitored until all mice started to gain weight (~12 dpi). After challenge with the PRVABC59 strain 6 weeks after first infection with 10^6^ plaque forming units (PFU)/mouse in a volume of 100 μl. Mice were bled 3 weeks after heterotypic challenge for serum antibody.

### Viral RNA detection

Blood samples were allowed to clot at room temperature for 30 min and were then centrifuged for 4 min at 4 °C and 6000 rcf. Serum was separated from the clot and stored in a sterile microcentrifuge tube at −80 °C until further processing. Eye-swab samples were processed by adding 250 μl of M199E media to the swab cotton in a centrifuge tube, vortexing and centrifuging for 2 min at 4 °C and 6000 rcf. The supernatant was extracted and stored in a separate microcentrifuge tube at −80 °C. RNA extraction was performed using the Kingfisher^TM^ (Thermo-Fisher) automated extraction platform and the Ambion MagMax^TM^ viral isolation kit (Thermo-Fisher), as per manufacturer’s instructions. Viral RNA was detected by qRT-PCR using the SuperScript™ III One-Step RT-PCR System with Platinum® Taq DNA Polymerase (Life Technologies) on a Roche Lightcycler® 480 (Roche). ZIKV-specific primers and probe were previously designed by Faye et al. [20].

### Plaque reduction neutralization test (PRNT)

PRNTs were performed following the WHO PRNT protocol for dengue (DENV) which we have previously shown to be applicable to ZIKV [21, 22] on serum collected from surviving ZIKV-infected mice. Briefly, virus was standardized to 25 PFU of ZIKV in 50 μl per well. Mouse serum was complement inactivated for 30 min at 56 °C and then serially diluted 1:2 in M199E media from 1:10 to 1:1280 and mixed with 50 μl of the standardized virus solution and allowed to incubate at 37 °C for 1 h. The mixture was then inoculated onto ~80% confluent Vero cell monolayers in 12-well plates. Also included were a negative control well (media only) and a positive control (virus only). The first overlay was added immediately after incubation, and the second overlay was added 3 dpi for ZIKV. Plaques were visible and counted the following day (4 dpi). The percent reduction in plaques per dilution was calculated. When a negative plaque reduction was observed, the value was set at a baseline of zero. Results are expressed as the reciprocal of the dilution in which the desired percentage of plaque reduction was achieved; we report both PRNT50 and PRNT80.

### Statistics

Statistics were performed using SAS 9.4 (Cary, NC) or RStudio with R version 3.2.2. The changes in weight were reported as the percent lost compared to initial weight. Viremia was logarithmically transformed and reported as log10 titer. We evaluated the differences in weight and viremia between sexes and day post infection with a repeated measures ANOVA analysis, using a mixed model to evaluate the effect of sex, day post-infection, and the interaction of each on the percent weight reduction or the log viremia titer (SAS, PROC MIXED). As our sampling regimen was temporally unevenly spaced, we specified a spatial power covariance structure to account for the uneven intervals. For the heterotypic challenge experiment, PRNT50 and PRNT80 titers were expressed as the reciprocal of the highest dilution at which the desired percentage of plaque reduction was achieved. Differences in percent reduction of neutralization were analyzed using the Kruskal-Wallis rank sum test and the 95% confidence interval calculated from a t-distribution (df = 5). Figures were created with R version 3.2.2 or histologic images compiled in Microsoft PowerPoint (Seattle, WA).

## Results

### Infection kinetics of ZIKV in IRF3/7 DKO mice

**Fig. 1:**
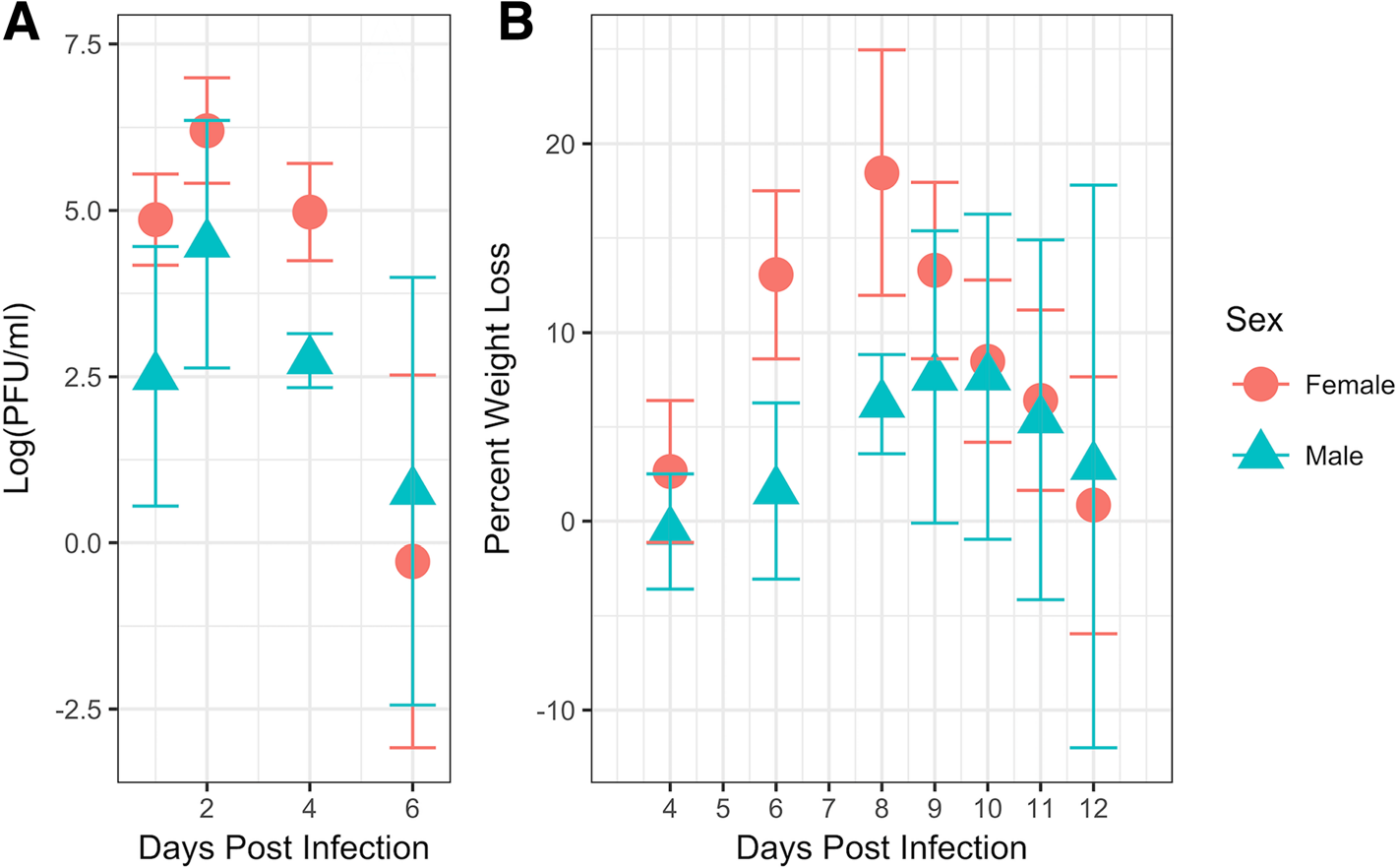
Repeated measures ANOVA analysis of ZIKV infected females (*red dots*) and males (*green triangles*) showing the average measurements for each group and time point, together with the 95% confidence interval. No significant difference was found in **a** log viral titer (RNA copies/ml) and **b** percent weight loss after infection with ZIKV

To test the susceptibility of IRF 3/7 DKO mice to ZIKV, we inoculated 10^6^ PFU/mouse of MR766 ZIKV Uganda strain subcutaneously in mice between 6 and 10 weeks of age, one group of males and one group of females. Viral RNA was detected in the serum of all mice, peaking at 2 dpi for both groups. The female mice had on average higher serum viral titers each day, with a peak average log titer of 6.2 compared to 4.49 in males, although this difference was not found to be significant (Fig. 1a, *p* > .05). Both groups of mice began to lose weight within 2 days of infection. The mice had a maximum average weight loss of 7.6% at 9 dpi for the males and 18.4% at 8 dpi for the females. Weight loss was not significantly different between sexes (Fig. 1b, *p* < .05). One male died at 7 dpi and two females had to be euthanized on 8 dpi because they lost over 21% of their initial body weight. This translates to an approximate 72% survival rate. The male mouse that died had a higher log titer than the average of the surviving male mice (log titer of 6.1 versus 4.39 of survivors). The two euthanized females had the two highest peak viral titers of the group (log titer of 7.24 and 6.58 versus the average of 5.84 of the other 4 females), although another female of the group had log titer of 6.53 and a peak weight loss of 16.3% (the second highest weight loss), but survived infection.

Signs of overt disease observed in all infected mice included reduced activity, hunched posture, and ruffled fur. These signs began around 5 dpi and increased in severity concurrently with weight lost, reaching a maximum at 8–9 dpi, at which point they began to regain weight and recover. In addition to the non-specific signs, two mice developed ocular disease in the form of crusty discharge in the eye: one male mouse in the right eye at 11 dpi, and one female mouse in the left eye at 6 dpi. The male mouse fully recovered as of 7 months post infection. The female mouse was euthanized at 8 dpi due to excessive weight loss. The ocular discharge was collected and tested for presence of ZIKV RNA, and found in the case of the female mouse to contain 2.280x10^3^ genome copies/mL of ZIKV NS5 as per the qPCR assay. Attempts to isolate infectious virus from the supernatant were unsuccessful.

### Histologic lesions

**Fig. 2:**
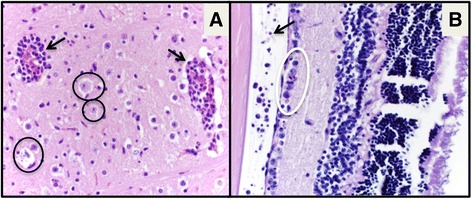
**a** Encephalitis, with perivascular cuffing as the result of recruitment of leukocytes into the brain (*arrows*) and neuronal degeneration and necrosis (*circled*) in a female ZIKV-infected mouse (400X magnification). **b** Ocular histology of a ZIKV-infected female mouse with ocular discharge and crusting around the eye that revealed retinal ganglionar cell necrosis (*circled*) and vitreitis with cellular infiltrate into what is supposed to be clear vitreous humor (*arrow*) at (400X magnification)

**Fig. 3:**
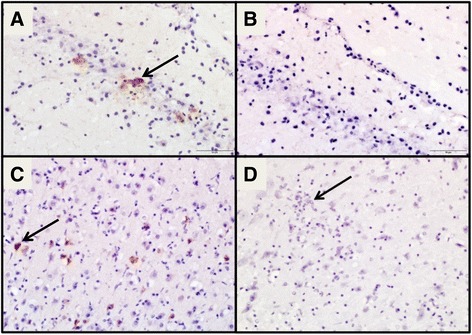
IHC labeling positive for ZIKV antigen in the brain (at *arrows*): **a** hippocampus of a ZIKV- infected female mouse and **c** cerebral cortex of the ZIKV- infected male mouse that died versus negative controls of **b** hippocampus and **d** cerebral cortex with evidence of encephalitis (*arrow*) (all at 400X magnification)

To explore the lesions produced by acute infection with ZIKV in IRF 3/7 DKO mice, we infected a second group of IRF3/7 DKO females with the purpose of analyzing their tissues histologically. The viremia in this second group was not statistically different from the other groups (Additional file 1: Figure S1, *p* > .05). We observed multifocal mild to moderate histologic lesions in the brain, spinal cord and eye of the sacrificed females. Two out of five female mice had encephalomyelitis with lymphocytic perivascular cuffing, gliosis and neuronal necrosis (Fig. 2a). Using IHC, we were able to visualize viral antigen in the hippocampus (Fig. 3a-b) of one of these females. Another female mouse from this group had bilateral epiphora and crusting at 7 dpi; at the time of euthanasia at 12 dpi, it had retinal ganglion cell necrosis and vitreitis, presenting inflammatory cell infiltration of the vitreous humor where in normal circumstances there is none (Fig. 2b).

In addition to these females, we also evaluated histologically the tissues of two mice from the infection kinetics groups: the one male mouse that died and the eyes of the female with ocular discharge that had to be euthanized. In spite of finding ZIKV RNA in the ocular discharge from this female, no histological lesions were found in either the sclera or the retina. The male mouse presented viral antigen visualized by IHC in the in the cerebral cortex (Fig. 3c–d), as well as in the male reproductive organs.

**Fig. 4:**
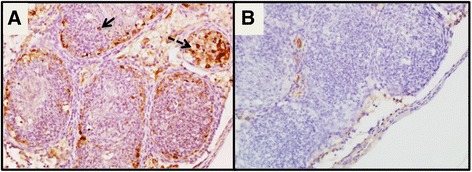
**a** IHC and hematoxylin of ZIKV-infected male mouse that died with necrosuppurative epididymitis with ZIKV antigen within the cytoplasm of lining epithelial cells (*solid arrow*) and sloughed intraluminal degenerate and necrotic epithelial cells (*dashed arrow*). **b** Negative control of the epididymis with necrosuppurative epididymitis (400X)

**Fig. 5:**
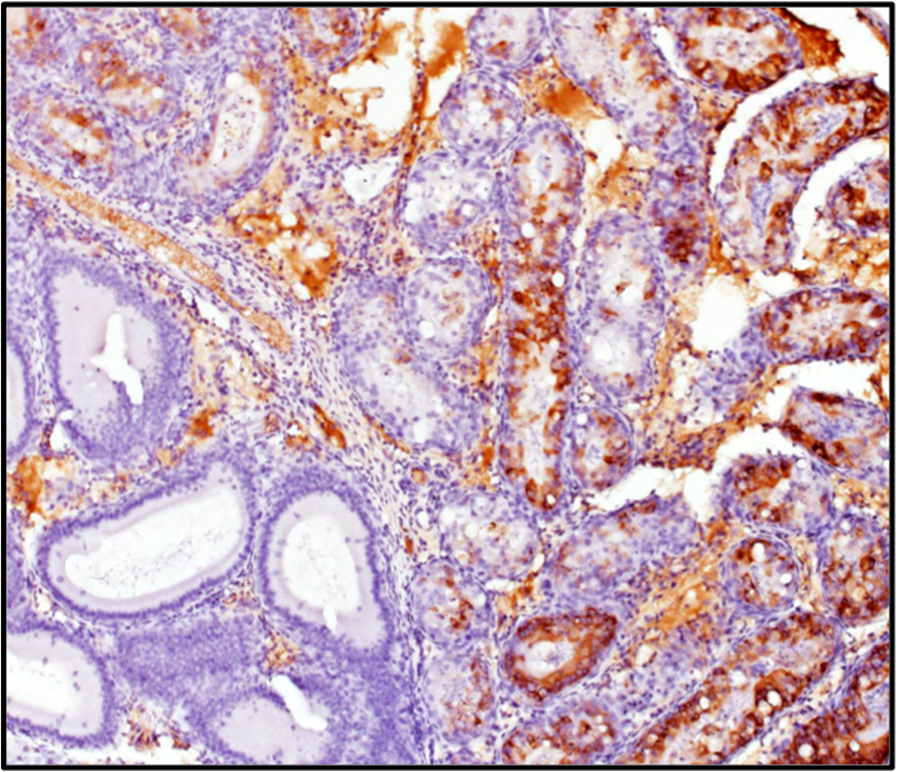
IHC from infected male that died of ZIKV shows extensive degeneration and necrosis of the epithelial lining associated with abundant viral antigen in the right-hand portion of the image (i.e. at *arrow*) compared to the internal negative control that shows a lack of viral antigen and intact normal tissue on the left (100X magnification)

**Fig. 6:**
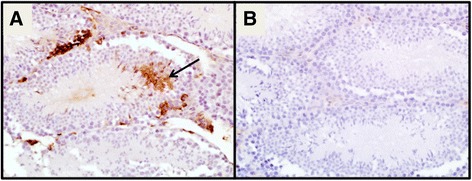
IHC shows labeling for ZIKV antigen in the germ cells of the seminiferous tubules in the testes at arrows (400X) (**a**) as opposed to the negative control (400X) (**b**)

**Fig. 7:**
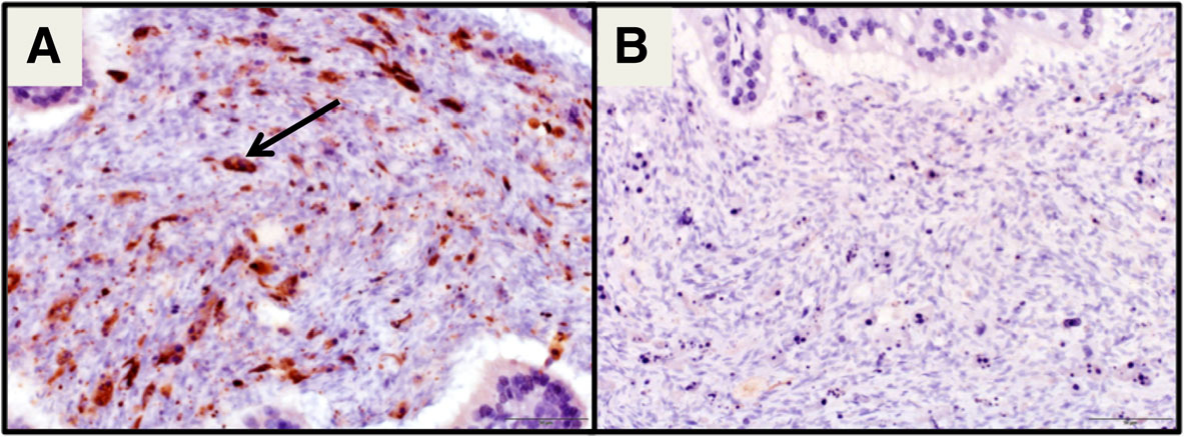
IHC shows ZIKV antigen within the seminal fluid in the lumen of the ductus deferens at arrows (400X) (**a**) versus negative control (400X) (**b**)

The reproductive organs of the male mouse presented severe necrosuppurative epididymitis associated with abundant viral antigen within the epididymal lining and sloughed off epithelial cells in the lumen (Fig. 4a and b). The affected portion of the epididymis was in stark contrast to other portions of the epididymis with normal architecture and no viral antigen (Fig. 5). Viral antigen was also visualized in germ cells in the seminiferous tubules of the testicles (Fig. 6). In addition, we observed abundant ZIKV antigen staining in the seminal fluid inside the lumen of the ductus deferens, mostly concentrated in what were interpreted as sloughed off epithelial cells (Fig. 7).

No lesions or viral antigen were found in the reproductive organs of the females, nor in skull bones, vertebrae, bone marrow, nerves, spinal root and trigeminal ganglia, upper respiratory tract, lungs, salivary glands, tonsils, spleen, lymph nodes, kidneys, adrenal glands, oral cavity, esophagus, stomach, intestines, skeletal muscle, skin, and pituitary gland.

### Antibody production and cross-neutralization

We show that the IRF3/7 DKO model has similar ZIKV susceptibility characteristics as the other recent models, such as viremia and signs of disease, without being 100% lethal or necessitating humane euthanasia in the majority of cases [1, 2, 5–7]. Since this is a predominantly non-lethal model, we explored the utility of this model for studies in which the antibody responses would be an important endpoint.

**Fig. 8:**
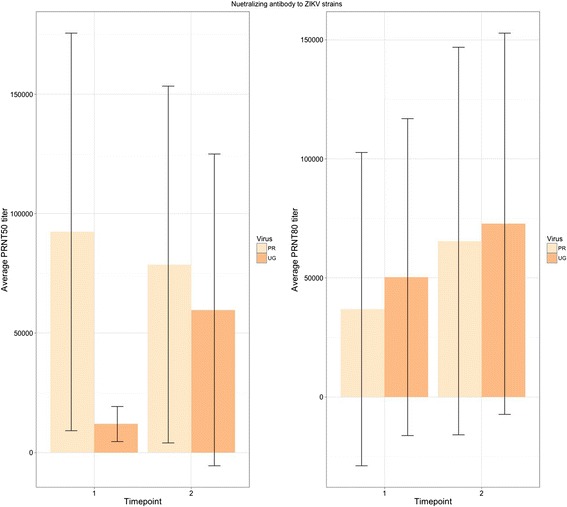
*Left*: Average PRNT50 titers for each ZIKV strain after primary (time point 1) and secondary (time point 2) challenges. *Right*: Average PRNT80 titers for each strain after primary (time point 1) and secondary (time point 2) challenges

**Fig. 9:**
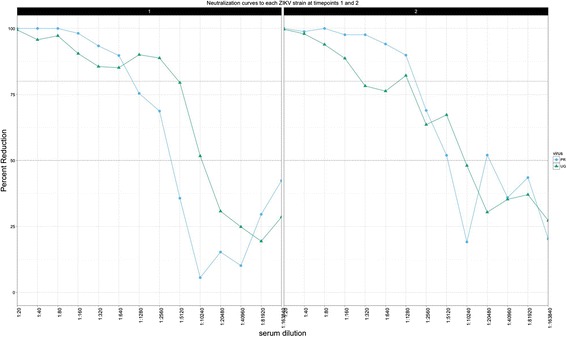
Top: Average percent plaque neutralization at time point 1 (*left*) and 2 (*right*) for each ZIKV strain, MR766 (*green triangles*) and PRVABC59 (*blue circles*)

A group of mice was infected with ZIKV MR766 presented with similar infection kinetics and signs of disease as the previous experimental groups (Additional file 2: Figure S2), and one mouse was euthanized at 8 dpi due to excessive weight loss. After the initial infection with the MR766 strain (time point 1), convalescent serum demonstrated a high capacity for neutralization to both the MR766 strain and the PRVABC59 strain from the current outbreak and of the Asian lineage. There was no significant difference in the neutralizing titers at both the 50 and 80% levels between virus strains (*p* > .05) (Fig. 8), indicating that in vitro the antibody response was cross-neutralizing between the two genotypes. When the neutralization curves of secondarily (PRVABC59) challenged mice against both ZIKV MR766 and PRVABC59 strains were compared, there was no significant difference in the percent reduction of plaques between strains or pre- and post-challenge (Fig. 9) (*p* > .05), indicating that secondary exposure to PRVABC59 did not result in a change in the neutralizing capacity of the antibodies for either strain. This recapitulates what others have demonstrated [23].

At three-weeks post-secondary challenge with PRVABC59, the average PRNT50 titer was approximately 59733 and 78720 for MR799 and PRVABC59, respectively. The PRNT80 titers were approximately 72747 and 65293 for MR799 and PRVABC59, respectively (Fig. 8). There was no significant difference at either neutralization level for the two strains (*p* > .05). In addition, there was no significant difference in PRNT50 or PRNT80 levels for either virus strain between time points, suggesting no altering effect of heterotypic challenge as demonstrated by this in vitro assay.

## Discussion

This IRF3/7 DKO mouse strain is susceptible to ZIKV and shows similar infection kinetics as the other recent models without being 100% lethal, thus allowing for long-term studies [1, 2, 5–7]. In addition, we demonstrate that this model of MR766 infection presents similar tissue tropisms and clinical presentation to those induced by strains of both African and Asian lineages in other models [4–6, 24, 25]. Specifically, we observed ocular discharge containing viral RNA, as well as infection in neurological and male reproductive tissues. The ocular involvement we observe in our mouse model corresponds to the increasing reports of ocular lesions in human patients associated to the strains of ZIKV circulating in the Americas, in both adult and congenital cases [26–30]. Adults often present with conjunctivitis, while in congenital cases the lesions commonly affect the chorioretinal region of the eye [28, 29, 31–34]. While we were unable to isolate viable virus from the ocular discharge of our infected mice, we did detect viral RNA, which has also been demonstrated in human conjunctival fluid [27].

We visualized the localization of ZIKV antigen in the male reproductive organs through IHC, observing labeling in the germ cells in the seminiferous tubules and the epithelial lining of the epididymis. In the epididymis, labeling was co-localized with abundant tissue destruction and inflammation in the form of necrosuppurative epididymitis, which could account for reports of hematospermia in a ZIKV-infected man in French Polynesia [35]. ZIKV antigen labeling of the seminal fluid in the lumen of the ductus deferens also suggests this model could support studies of sexual transmission. Importantly, immunohistochemical labeling of the sloughed epithelial cells from the epididymis for ZIKV could indicate alternate cellular mechanisms of sexual transmission in addition to spermatozoa [36]. The finding of epithelial cells containing ZIKV antigen in the seminal fluid of the mouse may also provide elucidation of sexually transmitted ZIKV in a vasectomized male where sperm cells would not have comprised a potential source for viral transfer during intercourse [37].

Interestingly, signs of infection (hunched posture, inactivity) and mortality were most severe around 8–9 dpi when viremia was long past peak at 2 dpi. By 6dpi, the clearance of systemic viremia was well underway, with only 3 mice presenting detectable quantities of virus RNA in serum. It is likely that at 8–9 dpi the virus was practically absent from the serum [1], and this is supported by observations in other mouse models where over the first 6 days of infection the amount of viral RNA in serum decreases, but increases in the testes and brain [6]. Upon sacrifice at 7 days post infection, mice showed signs of infection-induced lesions in several organs. This suggests that severe clinical signs occur at the time of viral dissemination to the organs rather than at the time of peak viremia. If human patients present a similar timing of symptom onset, by the time patients present to the clinic with rash or other signs of infection, the systemic viremia may be post-peak and, in extreme cases, absent. We demonstrated that virus is still found in tissues when serum viremia titers are low, and this may explain the enhanced detection capabilities in human urine samples up to 2 weeks post overt infection and in semen up to 93 days post symptoms [38–40].

Though some reports have demonstrated that plaque neutralization results do not correlate with protection [41], our study replicates what was previously shown in a similar mouse model [23] to confirm that infection with either the African or Asian strains of ZIKV is enough to produce cross-neutralizing antibody responses. Given the evidence of seroconversion in African populations, it is likely that the African lineage of ZIKV still circulates in Africa, but is underreported [42]. With the recent importation of the Asian lineage to Cape Verde from the current outbreak in the Americas and the promising developments of a ZIKV vaccine, there is a need to understand the interplay between these two genotypes using gold standard assays like the PRNT [43, 44]. Our results offer further evidence to support a single serotype of ZIKV with cross-protection among heterotypic lineages, and lends confidence to development of monovalent vaccine candidates.

The findings described herein characterize the IRF3/7 DKO mouse model for use in ZIKV infection studies. This model has the specific benefits of low mortality and robust antibody responses – comparable to the antibody response observed in a rhesus macaque model [7] – for studies in which antibody production is an important outcome. In summary, we find that this African strain of ZIKV is capable of causing lesions and disseminating to tissues, such as brain and testicles. This represents a similarly to what has been observed in the current outbreak where there have been observed human cases of neurological disease and involvement of male reproductive organs [31, 45, 46].

## Conclusions

Diversity of small animal models provides researchers with a varied set of tools with which to ask specific questions regarding ZIKV pathogenesis and transmission, as well as for investigations into treatment options. We offer here another such small animal model for evaluation of the pathogenesis of the ZIKV with opportunities for therapeutic evaluation, as antibody responses are critical for quantifying the efficacy of vaccine and other anti-viral candidates. Understanding the specific utility of various small animal models provides researchers with the tools with which to ask targeted questions regarding ZIKV pathogenesis, transmission, and treatment options.

## Additional files

Additional file 1: Figure S1. Repeated measures ANOVA analysis of females from different experimental groups demonstrated that no significant difference was found between the two groups 1) infection kinetics group (green triangles) and 2) histological lesions group (red dots). Points represent the average daily log PFU/ml for each group and the 95% confidence interval. (TIFF 2793 kb)

Additional file 2: Figure S2. Individual mice were bled every other day for 8 days after primary infection with the Ugandan MR766 ZIKV strain. Individual viremia (dots) and median (line) for each day for *n* = 3 mice daily. (JPG 42 kb)

## Abbreviations

CHIKV: Chikungunya virus
DENV: Dengue virus
DKO: Double knock out
DPI: Days post infection
IACUC: Institutional animal care and use committee
IFN: Interferon
IHC: Immunohistochemistry
IRF: Interferon regulatory factor
PFU: Plaque forming units
qRT-PCR: Quantitative, real-time PCR
ZIKV: Zika virus
