# Supplementary figures and images for: Tissue tropisms, infection kinetics, histologic lesions, and antibody response of the MR766 strain of Zika virus in a murine model
Source: Virol J. 2017 Apr 18;14:82. doi: 10.1186/s12985-017-0749-x (PMC5395720; doi:10.1186/s12985-017-0749-x)

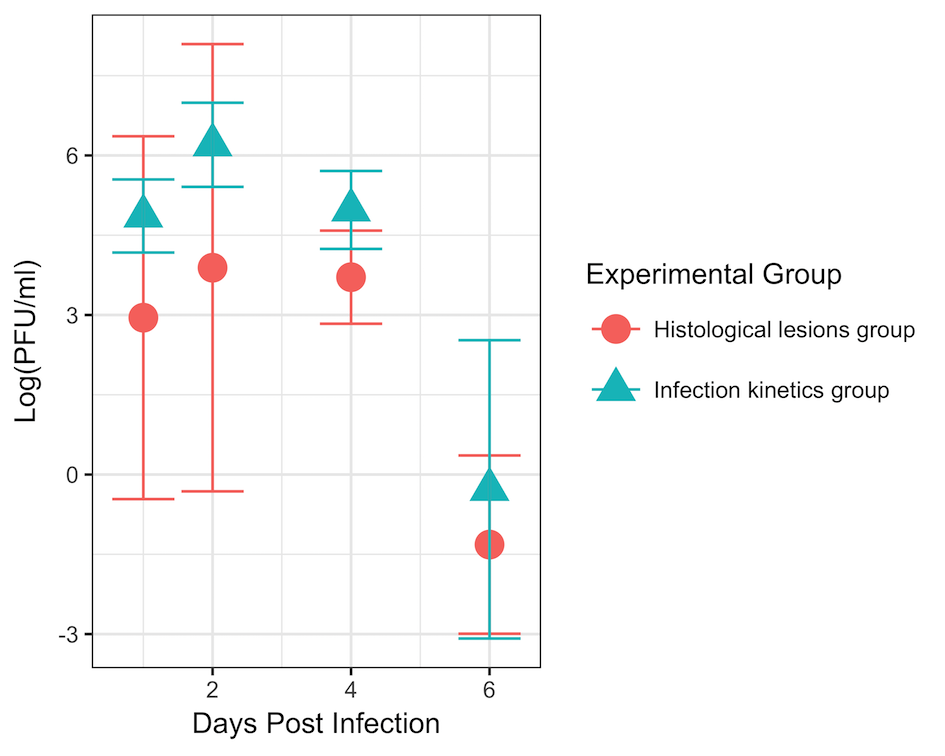

Supplement: Supplementary file 1 — Repeated measures ANOVA analysis of females from different experimental groups demonstrated that no significant difference was found between the two groups 1) infection kinetics group (green triangles) and 2) histological lesions group (red dots). Points represent the average daily log PFU/ml for each group and the 95% confidence interval. (TIFF 2793 kb) [file 12985_2017_749_MOESM1_ESM.tiff]

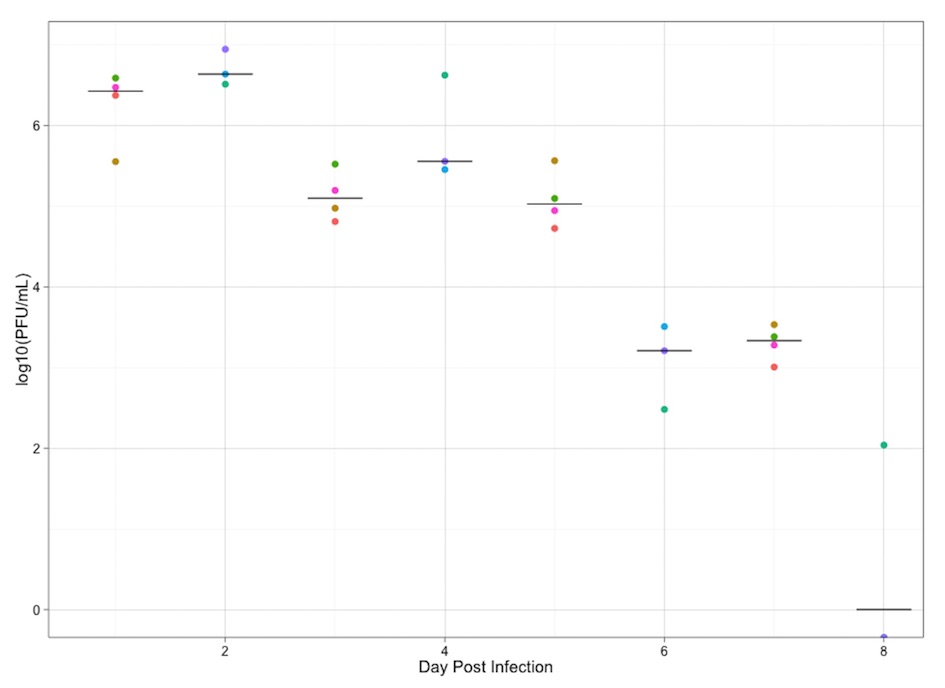

Supplement: Supplementary file 2 — Individual mice were bled every other day for 8 days after primary infection with the Ugandan MR766 ZIKV strain. Individual viremia (dots) and median (line) for each day for n = 3 mice daily. (JPG 42 kb) [file 12985_2017_749_MOESM2_ESM.jpg]
